# Growth modeling approach with the Verhulst coexistence dynamic properties for regulation purposes

**DOI:** 10.1007/s12064-023-00397-x

**Published:** 2023-07-08

**Authors:** A. J. Morales-Erosa, J. Reyes-Reyes, C. M. Astorga-Zaragoza, G. L. Osorio-Gordillo, C. D. García-Beltrán, G. Madrigal-Espinosa

**Affiliations:** Tecnológico Nacional de México, Centro Nacional de Investigación y Desarrollo Tecnológico, Interior Internado Palmira S/N, 61490 Cuernavaca, Morelos México

**Keywords:** Logistic, Growth, Coexistence, Coupling, Dynamic, Regulation

## Abstract

For this research, the properties of the logistic growth model for independent and coexisting species were used to set definitions for the possible regulation of one or two growth variables through their coupling parameters. The present analysis is done for the single-species Verhulst model without coupling, the single-species Verhulst model coupled with an exogenous signal, and the two-species Verhulst coexistence growth model which represents six different ecological regimes of interaction. The models’ parameters, such as the intrinsic growth rate and the coupling, are defined. Finally, the control results are expressed as lemmas for regulation, and they are shown using a simulation example of a fish population growing independent of human interaction (no harvesting, no fishing) and the simulation of the regulation of said population when the coupling of fish and humans is involved (harvesting, fishing).

## Introduction

The logistic or Verhulst-Pearl model was introduced to predict the population growth of New York in the USA (Pearl and Reed 1977); the model was stated to be too rigid and needed more flexibility, mainly because it has a fixed inflection point, thus creating always a symmetric curve in an *S*-shaped form. Since then, the model has been adapted to propose different single-species growth models able to reconfigure the *S* trajectory, such as the Richards, Von-Bertalanffy, and Tsoularis–Wallace growth models (Richards 1959; Von Bertalanffy 1957; Tsoularis and Wallace 2002). If the variable in question is increasing its value with delimited space and resources, then it can be modeled based on the logistic growth.

The logistic function is mostly used to model population dynamics, such as the growth of animals (Teleken et al. 2017; Brunner et al. 2019), plants (Birch 1999), seeds (Kocira 2018; Szparaga and Czerwińska 2017; Szparaga and Kocira 2018), microorganisms (Peleg et al. 2007), the spread of epidemic outbreaks (Wang et al. 2012; Bürger et al. 2019; Malavika et al. 2021; Aviv-Sharon and Aharoni 2020; Wu et al. 2020), and even related to the industry such as manufacturing and logistics population Zhou and Yan (2021). Some specific examples worth mentioning are the number of fruits of a certain plant under different conditions (Sari et al. 2019), the enzyme activity within the seeds during germination and early growth stages (Hämäläinen and Reinikainen 2007; Kuusela et al. 2004), and the size and number of fish population (Chaudhuri 1988; Laham et al. 2012).

The isolated growth of single species has a potential problem for its regulation given that it is not possible to decrease its growth, and the trajectory of the model has a fixed maximum value, which cannot be modified. However, when a single growing variable is coupled with an exogenous signal or with another population, the regulation of one of the said variables becomes possible.

The growth of two interacting populations can be described by coupling both variables based on the Verhulst and the Lokta–Volterra competition models, constructing a growth coexistence model (Ribeiro et al. 2014; Ribeiroa et al. 2010). Specific examples of this type of interaction found in the literature are the behavior of the human population under implemented policies (Cruz-Rivera and Vasilieva 2013) and under different ideologies (Vitanov et al. 2010), the growth of different species of fish (Rahman et al. 2006, 2008) and different species of turtles (Keevil et al. 2021), the growth of two types of breast cancer cells in tumors (Freischel et al. 2021), the growth of minor and mayor industry providers (Sundarakani et al. 2019), and the growth of vehicles with a different types of fuel (Sun and Wang 2018). The problem is not precisely the modeling of different processes, but what, when, and how the coupling of these variables is defined and in which instances can it be used for regulation. The regulation of this type of growth has been analyzed when an external source affects a parameter defined as the maximum carrying capacity which is related to the maximum growth (Cabella et al. 2012, 2010), whereas in this research, the external source is presented within the coupling parameters.

The objective of this research is to define the properties of the independent logistic growth, the logistic growth with a coupled exogenous signal, and the coexistence growth for modeling nonlinear dynamical systems and analyze their behavior and regime of competition. Furthermore, the definition of lemmas for the regulation of one and two populations is proposed using the coupling parameter.

## Logistic growth models preliminaries

### Verhulst model

The logistic or Verhulst-Pearl dynamic model can be used to reproduce the behavior of any variable increasing at an intrinsic growth rate from an initial condition to a maximum carrying capacity, and it is expressed with the following differential equation:

1 $$\begin{aligned} \dot{x}(t)=rx(t)\left( 1-\frac{x(t)}{N}\right) \hspace{0.5cm}, \hspace{1cm} x(0)=x_0 \end{aligned}$$

where $x(t)\in \mathbb {R}\ge 0$ is the instant value of the growth variable, $r\in \mathbb {R}\ge 0$ is the intrinsic growth rate, $N\in \mathbb {R^+}$ is the maximum carrying capacity, and $x_0$ is the initial condition. Expanding the right-hand side of (1) results in two terms, the first one is *rx*(*t*), which stands for the exponential positive growth rate of the variable (or population); this term can be linked to the birth rate; the second term is $-\tfrac{rx^2(t)}{N}$, and it represents the negative growth rate or the death rate. Whenever $x(t)=N$, the positive and the negative growth terms are canceled, the population growth rate stops, and the system is in equilibrium; therefore, if any manipulation is to be done, it has to be before the transitory duration finishes or the growing variable reaches its maximum carrying capacity.

The Verhulst or logistic model (1) has analytical solution, and the value of *x*(*t*) is given by

2 $$\begin{aligned} x(t)=\frac{x_0N}{(N-x_0)e^{-rt}+x_0}; \end{aligned}$$

Equation (2) is also known as the logistic growth function. The equilibrium points or the solutions of the Verhulst model when $\dot{x}(t)=0$ are defined:

#### Lemma 1

Let $r>0$ and $N>0$, then the growth model (1) has two equilibrium points $x^0$, the first one is trivial ($x^0_{T}$) and the second one is non-trivial ($x^0_{nT}$):

3 $$\begin{aligned} x^0_T&=0 \nonumber \\ x^0_{nT}&=N \end{aligned}$$

When *x*(*t*) is equal to either equilibrium point, its growth rate is zero. With Lemma 1, it can be established that to achieve a growing behavior, the initial condition of the model must be different from its equilibrium points, $x_0\ne x^0_{T}$ and $x_0\ne x^0_{nT}$. Using the analytical solution (2), the final value of the Verhulst model, denoted by $x^*$, is determined with:

#### Lemma 1.1

Let $x_0\ne x^0_T$, then the final value, $x^*$, of the trajectory of *x*(*t*) in (1) is equal to its maximum carrying capacity, expressed by

4 $$\begin{aligned} x^*=\lim _{t \rightarrow \infty }x(t)=N; \end{aligned}$$

for $x_0<N$, Eq. (1) grows positively toward *N*, and for $x_0>N$, it declines until reaching $x^*$. Note that the final value (4) is not dependent on the intrinsic growth rate *r*, and also the final value *x*(*t*) is equal to an equilibrium point. Between the initial condition and the final value, throughout the trajectory of the model, there could be an inflection point, $x_{ip}$, which is expressed as follows.

#### Lemma 1.2

Let $x_0\ne x^0_T$, then the trajectory of *x*(*t*) in model (1) has one inflection point, which value is half its maximum carrying capacity,

5 $$\begin{aligned} x_{ip}=\frac{N}{2} \end{aligned}$$

This means that the sigmoid trajectory created by the dynamic model has a symmetric behavior regarding $x_{ip}$. If $x_0\ge \tfrac{N}{2}$, then $x_{ip}$ does not exist and the growth of *x*(*t*) toward *N* is similar to the trajectory of a first-order linear differential equation.

For the simulation of the Verhulst model, information about its parameters must be known or measured. By assuming a value for *N*, information about the intrinsic growth rate *r* can be obtained from Lemmas 1.1 to 1.2 depending if the transitory time duration, or the time to inflection is known. Information can be extracted or estimated for an attempt to simulate and regulate models with structure as (1) in a time range of $0< t \le t_{ip}<t^*$, where $t_{ip}$ is the time to inflection and $t^*$ is the transitory time duration.

The **Transitory time duration** can be defined with

6 $$\begin{aligned} t^*= \left\{ \begin{aligned}&\frac{1}{r}\ln {\left( \frac{99(N-x_0)}{x_0}\right) }\hspace{0.45cm},\hspace{0.2cm}x_0<N\\&\frac{1}{r}\ln {\left( \frac{101(N-x_0)}{-x_0}\right) }\hspace{0.3cm},\hspace{0.2cm}x_0>N\\ \end{aligned} \right. \end{aligned}$$

Equation (6) is important because if the growth of the variable reaches its final value, the system’s dynamic is at equilibrium and the variable stops growing, so any change or regulation is only possible before $t=t^*$.

The **time to inflection** can be found before the transitory time duration ends, and it is the moment at which *x*(*t*) attains its maximum growth rate or when it is equal to the inflection point, defined with Lemma 1.2, and it can be expressed using the following equation:

7 $$\begin{aligned} t_{ip}= \begin{aligned} \frac{1}{r}&\ln {\left( \frac{(N-x_0)}{ x_0}\right) }\hspace{0.7cm},\hspace{0.2cm}x_0<x_{ip} \end{aligned} \end{aligned}$$

Using (7), the intrinsic growth rate can be computed before the process is finished; therefore, the creation of scenarios where some kind of regulation exists can be simulated. But given that the parameters and initial condition of the single-species logistic growth model are both positive and nonzero, its growth increases until reaching its maximum carrying capacity but it cannot decrease nor change its final value. Excluding a hypothetical manipulation of *N*, the final growth of the population cannot be regulated properly with the intrinsic growth rate *r*, and another variable has to be introduced.

### Verhulst model with coupling signal

If an exogenous signal, denoted by *v*, can affect the growth rate of a population, then the structure of the Verhulst model coupled with *v* can be expressed as:

8 $$\begin{aligned} \dot{x}(t)=rx(t)\left( 1-\frac{x(t)}{N}+\alpha \frac{v}{N}\right) ,\hspace{0.5cm} x(0)=x_{0} \end{aligned}$$

where $x(t)\in \mathbb {R}\ge 0$ is the instant value of the growth variable, $r\in \mathbb {R}\ge 0$ is the intrinsic growth rate, $N\in \mathbb {R^+}$ is the maximum carrying capacity, $\alpha \in \mathbb {R}$ is the coupling parameter, $0 < v \le \bar{v}$ is the exogenous signal, and $x_0$ is the initial condition.

The resulting terms of expanding the right-hand side of Eq. (8) have different interpretations since $\alpha$ can have positive or negative nature. The positive growth rate of (8) when $\alpha >0$ is $rx(t)+\tfrac{r\alpha x(t)}{N}v$, where *v* is an external source that benefits the growth rate, and the negative growth rate is $-\tfrac{rx^2(t)}{N}$. The other interpretation is when $\alpha <0$, then the positive growth rate is *rx*(*t*) and the negative growth rate becomes $-\tfrac{rx^2(t)}{N}+\tfrac{r\alpha x(t)}{N}v$, where *v* now inhibits the growth rate because of $\alpha$’s negative value. As it can be seen from the terms before, *v* with the coupling parameter directly affects the overall growth rate; therefore, this structure suggests a better regulation of the model.

The Verhulst model when coupled with *v* has analytical solution, the instant value of *x*(*t*) is

9 $$\begin{aligned} x(t)=\frac{x_{0}N+x_{0}\alpha v}{(N+\alpha v-x_{0})e^{-rt(1+\alpha v/N)}+x_{0}} \end{aligned}$$

The equilibrium points for model (8) are defined according to the following Lemma:

#### Lemma 2

Let $v \ne 0$ and $\vert \alpha v\vert <N$, then model (8) has the following equilibrium points:

10 $$\begin{aligned}&x^0_T=0 \nonumber \\&{x_{nT}^0}={N}+{\alpha v} \end{aligned}$$

When *v* attains its minimum value, then the model becomes the original single-species Verhulst model (1) which trivial and non-trivial equilibrium points are $x^0_T=0$ and $x^0_{nT}=N$, respectively, but whenever *v* attains a value different than zero, the solutions are defined with Lemma 2. The non-trivial equilibrium point is also the final value of the growth model coupled with an external signal, and $x^*$ is given as follows.

#### Lemma 2.1

Let $x_{0}\ne x^0_{T}$, $v\ne 0$ and $\vert \alpha v\vert <N$, the trajectory of *x*(*t*) in (8) achieves nonzero and a positive final value expressed as

11 $$\begin{aligned} x^*= \lim _{t \rightarrow \infty }{x}(t)={N}+{\alpha v} \end{aligned}$$

In (11), if $\alpha >0$, then $x^*$ is greater than the maximum carrying capacity, but if $\alpha <0$, then it is inferior to *N*. When $v=0$, the coupled model (8) turns into the one species Verhulst model which final value is *N*. Lemma 2.1 holds that the final value of the population or growth variable can be regulated through the coupling parameter, $\alpha$ and exogenous signal *v*. The inflection point of model (8) can be defined with this lemma:

#### Lemma 2.2

Let $x_{0}\ne x^0_{T}$, $v\ne 0$ and $\vert \alpha v\vert <N$, model (8) has a real and positive inflection point at

12 $$\begin{aligned} {x_{ip}}=\frac{1}{2}\left( N+\alpha v\right) . \end{aligned}$$

The inflection point (12) will be superior to half the maximum carrying capacity when $\alpha$ is positive, and $x_{ip}$ will be inferior to half the maximum carrying capacity when $\alpha$ is negative.

The **transitory time duration** of model (8), based on Lemma 2.1 and (9), is expressed with:

13 $$\begin{aligned} t^*= \left\{ \begin{aligned}&\frac{1}{r(1+\alpha v/N)}\ln {\left( \frac{99(N+\alpha v-x_{0})}{x_{0}}\right) }\hspace{0.5cm},\hspace{0.2cm}x_{0}<x^*\\&\frac{1}{r(1+\alpha v/N)}\ln {\left( \frac{101(N+\alpha v-x_{0})}{-x_{0}}\right) }\hspace{0.35cm},\hspace{0.2cm}x_{0}>x^*\\ \end{aligned} \right. \end{aligned}$$

And the **time to inflection**, based on Lemma 2.2, is expressed with

14 $$\begin{aligned} t_{ip}= \frac{1}{r(1+\alpha v/N)}\ln {\left( \frac{(N+\alpha v-x_0)}{ x_0}\right) }\hspace{0.95cm},\hspace{0.2cm}x_0<x_{ip} \end{aligned}$$

To solve Eqs. (13) and (14), the exogenous source *v* must be known and also the coupling parameter $\alpha$. When considering *v* as an external, added source, it is fairly assumed that this variable is known or measured, *v* can even be another known logistic growth function, and $\alpha$ can be seen as a manipulated variable for the regulation of the population.

### Verhulst coexistence model

The previous models consider one single population and its coupling with an exogenous source. The Verhulst coexistence model is used when two species are living together within the same space and shared resources. The model can have different ecological interactions depending on the nature of their coupling or competition parameters, i.e., when two different species stimulate each other’s growth the type of interaction is mutualism; when both species harm each other’s growth it is competition; if one of the species growth is independent and the other species growth gets stimulated or promoted, then it is commensalism; when the independent species growth harms or hinders the growth of the other, the interaction is amensalism; if one of the species is seen as the resource or food of the other species, then it is predation; when none of the species interact with each other, it is neutralism. A system of coexistence of two variables, denoted by $\Sigma _{CE}$, is expressed below:

15 $$\begin{aligned} \Sigma _{CE}: \left\{ \begin{array}{l} \dot{x}_{1}(t)=r_{1}x_{1}\left( 1-\frac{x_{1}(t)}{N_1}+\alpha _1\frac{{x}_{2}(t)}{N_{1}}\right) \\ \\ \dot{x}_{2}(t)=r_{2}x_{2}\left( 1-\frac{x_{2}(t)}{N_2}+\alpha _2\frac{{x}_{1}(t)}{N_{2}}\right) \end{array} \right. , \ \begin{array}{l} x_1(0)=x_{01} \\ \\ x_2(0)=x_{02} \end{array} \end{aligned}$$

where $x_{1}(t), x_{2}(t)\in \mathbb {R}\ge 0$ are the instant values of the growing variables, $r_{1}, r_{2}\in \mathbb {R}\ge 0$ are the intrinsic growth rates, $N_{1}, N_2\in \mathbb {R}^+$ are the maximum carrying capacities, $\alpha _{1}, \alpha _2 \in \mathbb {R}$ are the coupling or competition parameters, and $x_{01}$, $x_{02}$ are the initial conditions.

The product of the coupling or interspecific competition parameters is related to the type of coexistence between the species, as seen in Table 1. Moreover, the manipulation of these parameters not only can be used to regulate the growth of the variables, but also to control the ecological regime they represent.

**Table 1:** Type of coexistence based on the product of $\alpha _1\alpha _2$

Ecological regime	Coupling configuration	$\alpha _1\alpha _2$
Neutralism	$\alpha _1=0$ ,$\alpha _2=0$	$\alpha _1\alpha _2=0$
Commensalism	$\alpha _1=0$ ,$\alpha _2>0$	
	$\alpha _1>0$ ,$\alpha _2=0$	
Amensalism	$\alpha _1=0$ ,$\alpha _2<0$	
	$\alpha _1<0$ ,$\alpha _2=0$	
Mutualism	$\alpha _1>0$ , $\alpha _2>0$	$\alpha _1\alpha _2>0$
Competition	$\alpha _1<0$ , $\alpha _2<0$	
Predation	$\alpha _1<0$ , $\alpha _2>0$	$\alpha _1\alpha _2<0$
	$\alpha _1>0$ , $\alpha _2<0$	

The equilibrium points of the coexistence of two species consist of a pair of trivial solutions, i.e., ${x_{1T}^0}=0$ and ${x_{2T}^0}=0$, and another pair of non-trivial solutions given by the next lemma.

#### Lemma 3

Let $\alpha _{1}\alpha _{2}\ne 1$, and $x_{0j}> 0$ for $j=1,2;$, then model (15) has the next non-trivial equilibrium points ($x_{nT}^0$):

16 $$\begin{aligned} \left[ \begin{array}{c} x^0_{nT1}\\ \\ x^0_{nT2} \end{array}\right] =\left[ \begin{array}{cc} \frac{1}{1-\alpha _{1}\alpha _{1}}&{}\frac{\alpha _{1}}{1-\alpha _{1}\alpha _{2}}\\ \\ \frac{\alpha _{2}}{1-\alpha _{1}\alpha _{2}}&{} \frac{1}{1-\alpha _{1}\alpha _{2}} \end{array}\right] \left[ \begin{array}{c} N_{1}\\ \\ N_{2} \end{array}\right] \end{aligned}$$

Similarly to the previous models’ analysis, the final value of the growing species is the same as its respective non-trivial equilibrium point, this is expressed with Lemma 3.1

#### Lemma 3.1

Let $\alpha _{1}\alpha _{2}\ne 1$, and $x_{0j}>0$ for $j=1,2;$ then, the final value of the trajectory of each population has the following expression:

17 $$\begin{aligned} \lim _{t\rightarrow \infty }\left[ \begin{array}{c} x_{1}(t)\\ \\ x_{2}(t) \end{array}\right] =\left[ \begin{array}{cc} \frac{1}{1-\alpha _{1}\alpha _{2}}&{}\frac{\alpha _{2}}{1-\alpha _{1}\alpha _{2}}\\ \\ \frac{\alpha _{1}}{1-\alpha _{1}\alpha _{2}}&{} \frac{1}{1-\alpha _{1}\alpha _{2}} \end{array}\right] \left[ \begin{array}{c} N_{1}\\ \\ N_{2} \end{array}\right] \end{aligned}$$

And finally, the inflection points of the coexistence model are defined with

#### Lemma 3.2

If $\alpha _{1}\alpha _{2}\ne 1$, and $x_{0j}>0$ for $j=1,2;$, then, (8) has the following inflexion points:

18 $$\begin{aligned} \left[ \begin{array}{c} x_{ip1}\\ \\ x_{ip2} \end{array}\right] =\left[ \begin{array}{cc} \frac{2}{4-\alpha _{1}\alpha _{1}}&{}\frac{\alpha _{1}}{4-\alpha _{1}\alpha _{2}}\\ \\ \frac{\alpha _{2}}{4-\alpha _{1}\alpha _{2}}&{} \frac{2}{4-\alpha _{1}\alpha _{2}} \end{array}\right] \left[ \begin{array}{c} N_{1}\\ \\ N_{2} \end{array}\right] \end{aligned}$$

The equilibrium points, final value, and inflection points of the coexistence growth model are dependent on the coupling parameters, $\alpha _1$, and $\alpha _2$, as stated; this model represents six different ecological regimes with nine configurations, which means that Eqs. (16)–(18) are different for each of those configurations.

#### Coexistence configurations

This subsection is devoted to the analysis of the coexistence configurations that model (15) can achieve and the characterization of their coupling or competition parameters for each of the ecological regimes: neutralism, commensalism, amensalism, mutualism, competition, and predation.

**Neutralism** is the first configuration, it is a trivial one, achieved when

19 $$\begin{aligned} \alpha _{1}=0, \hspace{1cm} \alpha _{2}=0; \end{aligned}$$

as a consequence, the competition dynamic system in (15) becomes one without interaction of species. And since such configuration decouples the dynamic of the species, the equilibrium points, the final value, and the inflection point of each of the trajectories follow Lemmas 1–1.2.

**Commensalism** is the type of coexistence for the second and third configurations. This is achieved when one parameter is positive and the other one is zero. If the species denoted by 1 has an independent growth ($\alpha _{1}=0$), and the one denoted by 2 takes advantage of species 1 or has a positive competition parameter ($\alpha _{2}>0$), then the corresponding structure of model (15) is,

20 $$\begin{aligned} \Sigma _{CA12}: \left\{ \begin{array}{l} \dot{x}_{1}(t)=r_{1}x_{1}\left( 1-\frac{x_{1}(t)}{N_1}\right) \\ \\ \dot{x}_{2}(t)=r_{2}x_{2}\left( 1-\frac{x_{2}(t)}{N_2}+\alpha _2\frac{{x}_{1}(t)}{N_{2}}\right) \end{array} \right. \end{aligned}$$

where $\dot{x}_1(t)$ has the properties of the single-species Verhulst growth model (Sect. 2.1), but for $\dot{x}_2(t)$, Lemma 3 defines the equilibrium points, Lemma 3.1 the final value and Lemma 3.2 the inflection point. If the species denoted by 2 has an independent growth ($\alpha _{2}=0$), and the one denoted by 1 benefits from species 2 ($\alpha _{1}>0$), then the model structure is,

21 $$\begin{aligned} \Sigma _{CA21}: \left\{ \begin{array}{l} \dot{x}_{1}(t)=r_{1}x_{1}\left( 1-\frac{x_{1}(t)}{N_1}+\alpha _1\frac{{x}_{2}(t)}{N_{1}}\right) \\ \\ \dot{x}_{2}(t)=r_{2}x_{2}\left( 1-\frac{x_{2}(t)}{N_2}\right) \end{array} \right. \end{aligned}$$

where Lemmas 3–3.2 now hold for $\dot{x}_1(t)$ and $\dot{x}_2(t)$ has the properties of the single species Verhulst growth model.

**Amensalism** is the contrary situation and the fourth and fifth configurations, which are achieved when one coupling parameter is negative and the other one is zero. For $\alpha _{1}=0$ and $\alpha _2<0$, the system’s structure is the same as (20), species number 1 follows Lemmas 1–1.2; meanwhile, species number 2 follows Lemmas 3–3.2 as long as $\alpha _{2} \ge -\frac{N_{2}}{N_{1}}$. For $\alpha _1<0$ and $\alpha _2=0$, the structure coincides with (21), where Lemmas 3–3.2 hold true for species 1 as long as $\alpha _{1}\ge -\frac{N_{1}}{N_{2}}$ and species 2 follows Lemmas 1–1.2.

**Mutualism** is the sixth configuration, achieved when model (15) has positive coupling or competition, i.e.,

22 $$\begin{aligned} \alpha _{1}>0, \hspace{1cm}\alpha _{2}>0, \end{aligned}$$

and if the following inequality is fulfilled,

23 $$\begin{aligned} 0<\alpha _1\alpha _2<1; \end{aligned}$$

then, the structure of the coexistence model is as (15), and its equilibrium points are defined by Lemma 3, final values by Lemma 3.1 and inflection points by Lemma 3.2. From those definitions, it can be seen that if $\alpha _1\alpha _2=1$, then there is no real solution. Besides, if the product $\alpha _1\alpha _2>1$, then the system becomes unstable, the nontrivial equilibrium point, the final value, nor the inflection point are ever reached.

**Competition** is the contrary of mutualism, and it is the seventh configuration. This is achieved when the coupling parameters of (15) are both negative,

24 $$\begin{aligned} \alpha _{1}<0, \hspace{1cm}\alpha _{2}<0, \end{aligned}$$

where Lemmas 3–3.2 also held true for both of the species. For this regime to have a real solution, then $\alpha _1\alpha _2\ne 1$, nevertheless, the system does not become unstable for $\alpha _1\alpha _2\ne >1$ but this means the extinction of one of the species, populations, or growth variables.

**Predation** represents the eighth and ninth configurations; this is for prey-predator interactions. For this to be achieved, the competition parameter associated with the prey must be negative and the one associated with the predator must be positive. Lemmas 3–3.2 are true for this type of interaction; however, to achieve non-negative values, the next conditions are to be fulfilled. If the predator population is denoted by 1, the coupling parameters have to achieve the following inequalities

25 $$\begin{aligned} \alpha _{1}>0, \hspace{3mm} -\frac{N_{1}}{N_{2}}\le \alpha _{2}<0, \end{aligned}$$

and when the predator species population is denoted by 2, the inequalities that must be held for $\alpha _{1,2}$ are

26 $$\begin{aligned} -\frac{N_{2}}{N_{1}}\le \alpha _{1}<0, \hspace{3mm} \alpha _{2}>0. \end{aligned}$$

When negative final values are obtained, or when inequalities (25) or (26) are not fulfilled, it means that the predator population exterminates the prey population.

## Regulation of logistic growth models

### Final value configurations

The final value (11) of the model (8), which is coupled with an external signal, $0<v\le \bar{v}$, can be rewritten considering $\alpha$’s configuration as follows:

27 $$\begin{aligned} \lim _{t \rightarrow \infty }{x}(t)= \left\{ \begin{array}{l} {N}+{\alpha v}\\ \\ 0 \end{array} \right. \hspace{0.2cm},\hspace{0.2cm} \ \begin{array}{l} -\frac{N}{v}<\alpha \\ \\ \alpha \le -\frac{N}{v}\hspace{0.1cm} \end{array} \end{aligned}$$

The regulation of the final value through the coupled parameter is proposed from the previous equation. If $\alpha >0$, the growth’s final value is defined but it cannot be regulated because of the positive configuration of the parameter. If $\alpha$ is established as negative or zero, i.e., $-\frac{N}{v}<\alpha \le 0$, the growth can be regulated between zero and *N*, because for $\alpha =0$, the final value is that of the classic logistic Verhulst model, and for $\alpha =-N/v$, the growth’s final value is zero. When this is possible, $\alpha$ can be defined for regulation with the following lemma:

#### Lemma 4

Let the control error be $e:=x(t)-x_d$ and the manipulated variable be $\alpha :=\alpha (t)<0$. Then, the final value of the population *x*(*t*) can be regulated using the following control law:

28 $$\begin{aligned} \alpha (t)=\frac{1}{v}\left( -\frac{KN}{rx(t)}({x(t)-x_d})-{N}+{x(t)}\right) \end{aligned}$$

This ensures the final value of *x*(*t*) is equal to the desired population, $x_d$, for positive values of the gain, *K*.

$$\lim _{t \rightarrow \infty }{x}(t)=N+\alpha (t)v=x_d$$

The same analysis is done for the coexistence model, (15), their final values can be expressed differently depending on the regime’s configuration, and a general representation of all of them is shown as follows:

29 $$\begin{aligned} \lim _{t \rightarrow \infty }{x_j}(t)= \left\{ \begin{array}{l} \infty \\ \\ \frac{{N_j}+{\alpha _j N_i}}{1-\alpha _j\alpha _i}\\ \\ \frac{{N_j}+{\alpha _j N_i}}{1-\alpha _j\alpha _i}\\ \\ 0 \end{array} \right. \hspace{0.1cm},\hspace{0.1cm} \ \begin{array}{l} \alpha _j>0 \hspace{0.1cm}\wedge \hspace{0.1cm} \alpha _j\alpha _i\ge 1\\ \\ \alpha _j>0 \hspace{0.1cm}\wedge \hspace{0.1cm} \alpha _j\alpha _i<1 \\ \\ -\tfrac{N_j}{N_i}<\alpha _j\le 0\hspace{0.1cm}\wedge \hspace{0.1cm} \alpha _i\alpha _j<1 \\ \\ \alpha _j \le -\tfrac{N_j}{N_i}\hspace{0.1cm} \end{array} \end{aligned}$$

The following remarks are made regarding the conditions for control over this kind of mathematical model (15):

#### Remark 1

From Eq. (29), it is noted that the population can reach a state of overgrowth when its coupling parameter is positive $\alpha _{j}>0$. But this (overgrowth) becomes unstable when $\alpha _j \alpha _i\ge 1$, and its final value is never reached.

By looking at the results from Sect. 3.3.1, the coexistence configurations that have these conditions are: Commensalism, which final values will be,

$$\begin{aligned} \lim _{t\rightarrow \infty }\left[ \begin{array}{c} x_{1}(t)\\ \\ x_{2}(t) \end{array}\right] = \left[ {\begin{array}{l} {{N_1}+{\alpha _1 N_2}}\\ \\ {N_2} \end{array}}\right] \hspace{0.1cm},\hspace{0.1cm} \ \begin{array}{l} \alpha _1>0 \\ \\ \alpha _2=0 \end{array} \end{aligned}$$

and Mutualism, the overgrowth for this regime becomes unstable when $\alpha _j \alpha _i\ge 1$, but if this inequality holds, $0<\alpha _j \alpha _i< 1$, the final population’s growth of two species positively coupled is,

$$\begin{aligned} \lim _{t\rightarrow \infty }\left[ \begin{array}{c} x_{1}(t)\\ \\ x_{2}(t) \end{array}\right] =\left[ {\begin{array}{l} \frac{{N_1}+{\alpha _1 N_2}}{1-\alpha _1\alpha _2}\\ \\ \frac{{N_2}+{\alpha _2 N_1}}{1-\alpha _1\alpha _2} \end{array}}\right] \hspace{0.1cm},\hspace{0.1cm} \ \begin{array}{l} \alpha _1>0 \\ \\ \alpha _2>0 \end{array} \end{aligned}$$

Since $\alpha _1>0$ and $\alpha _2>0$, it is not possible to control these kinds of systems.

#### Remark 2

Notice from Eq. (29) that the regulation of the growth variable or population *j* is possible between $N_j$ and zero. Such regulation is accomplished when $-\tfrac{N_j}{N_i}<\alpha _j<0$.

The regimes of coexistence that can be regulated are: Amensalism, which have the following final values under said condition

$$\begin{aligned} \lim _{t\rightarrow \infty }\left[ \begin{array}{c} x_{1}(t)\\ \\ x_{2}(t) \end{array}\right] = \left[ {\begin{array}{l} {{N_1}+{\alpha _1 N_2}}\\ \\ {N_2} \end{array}}\right] \hspace{0.1cm},\hspace{0.1cm} \ \begin{array}{l} -\frac{N_1}{N_2}<\alpha _1\le 0 \\ \\ \alpha _2=0 \end{array} \end{aligned}$$

Competition, this regime has these final values,

$$\begin{aligned} \lim _{t\rightarrow \infty }\left[ \begin{array}{c} x_{1}(t)\\ \\ x_{2}(t) \end{array}\right] = \left[ {\begin{array}{l} \frac{{N_1}+{\alpha _1 N_2}}{1-\alpha _1\alpha _2}\\ \\ \frac{{N_2}+{\alpha _2 N_1}}{1-\alpha _1\alpha _2} \end{array}}\right] \hspace{0.1cm},\hspace{0.1cm} \ \begin{array}{l} -\frac{N_1}{N_2}<\alpha _1\le 0\\ \\ -\frac{N_2}{N_1}<\alpha _2\le 0\end{array} \end{aligned}$$

And Predation, where the regulation is specifically for the prey, has these final values,

$$\begin{aligned} \lim _{t\rightarrow \infty }\left[ \begin{array}{c} x_{1}(t)\\ \\ x_{2}(t) \end{array}\right] = \left[ {\begin{array}{l} \frac{{N_1}+{\alpha _1 N_2}}{1-\alpha _1\alpha _2}\\ \\ \frac{{N_2}+{\alpha _2 N_1}}{1-\alpha _1\alpha _2} \end{array}}\right] \hspace{0.1cm},\hspace{0.1cm} \ \begin{array}{l} \alpha _1>0 \\ \\ -\frac{N_2}{N_1}<\alpha _2\le 0 \end{array} \end{aligned}$$

From remark 2, a control law for the regulation of the regimes with at least one negative coupling parameter can be defined with a lemma such as:

#### Lemma 5

Let $e_j:=x_j(t)-x_{jd}$, where $x_{jd}$ is the desired final value, and $\alpha _j:=\alpha _j(t)$ subject to the condition $-\tfrac{N_j}{N_i}<\alpha _j(t)<0$, for $j=1,2$ and $i=3-j$. Then, the final value of $x_j(t)$ can be regulated using the following control law:

30 $$\begin{aligned} \alpha _j(t)=\frac{1}{x_i(t)}\left( -\frac{K_jN_je_j}{r_jx_j(t)}-{N_j}+{x_j(t)}\right) \end{aligned}$$

which ensures that the final value of the population equals the desired growth.

$$\begin{aligned} \lim _{t \rightarrow \infty }{x}_j(t)=x_{jd} \end{aligned}$$

#### Remark 3

Finally, from Eq. (29), it is noted that the population that has a negative coupling parameter can go to extinction. Therefore, the population’s final growth will be zero when $\alpha _{j}\le -\frac{N_j}{N_i}$ for $j=1,2$, $i=3-j$.

The types of coexistence where this condition is possible are Amensalism, Competition, and Predation. When this condition is met, the final value of the population coupled with a negative parameter will be zero. And when the weaker population gets extinct, the other variable, species, or population will grow without competition until it reaches its maximum carrying capacity.

## Example and application

For the application of the previous results, the following simulation example of a fish population is explained. For harvesting purposes, a fish population (*x*) grows under certain and regulated conditions regarding their resources and space, such that its intrinsic growth rate is $r=0.8$ and maximum carrying capacity is $N=780500$. Besides, it is known that at $t\approx 6$ months, the fish population without external manipulation reaches a maturity point such that the growth rate of the population starts declining (Laham et al. 2012). After 12 months of the fish population growing independently, a Fishing season begins with a duration of 6 months, when the fishing season is over, a new growing season begins which lasts for another 6 months, switching between seasons for a total duration of 60 months. The objective is to prevent the extinction of the fish population and if possible to regulate the population to the desired value for the fishing and growing seasons. The first simulation consists of the Fish population growing for 12 months without external interaction using the Verhulst model (1). The second simulation considers the interaction of the fish population with fishermen as an exogenous signal for the fishing season using the Verhulst model with coupling signal (8). And the third simulation considers the fishermen as another logistic growth equation, thus defining its coexistence with the fish population in the regime of Amensalism using model (15).

### Simulation of a single-species population

**Fig. 1:**
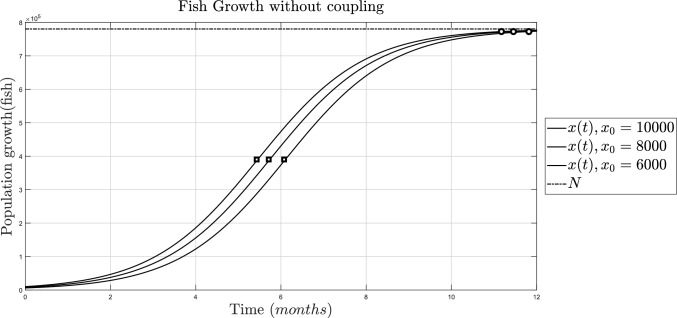
Simulation of the Verhulst model for fish growth with different initial conditions when there is no harvest ($\alpha =0$). For $x_0=1000$, the inflection point is reached at $t_{inf}=5.4$, represented by the square, and its final value at $t^*=11.2$, represented by the circle; for $x_0=800$, $x_{ip}$ is reached at $t_{inf}=5.6$ and $x^*$ at $t^*=11.4$; and for $x_0=6000$, $t_{inf}=6$ and $t^*=11.8$

With the information above, it is possible to simulate the growth of the fish population without any coupling, which can be reproduced with model (1). With the results in section 2.1, it is already known that the population will have two equilibrium points at $x=0$ and $x=780500$, the final value is at $x=780500$, and the inflection point is $x=390250$. It is also possible to assume that the maturity level the fish reach is their inflection point; therefore, the time to inflection can represent the moment when the fish are ready for harvesting, and the transitory time duration can represent the moment when the fish need to be harvested with urgency. Both of those properties can be calculated from Eqs. (6) and (7). The population simulation without harvest is done using the parameters from Table 2, and it is shown in Fig. 1.

**Table 2:** Parameters of the logistic growth model

Parameter	Value	Units
*r*	0.8	Fish/t
*N*	780500	Fish

Properties	Value	Units
$x^0_T$	$0 ^{1}$	Fish
$x^0_{nT}$	$780500 ^{1}$	Fish
$x^*$	$780500^{1}$	Fish
$x_{ip}$	$390250^{1}$	Fish

$^{1}$Properties were obtained from Lemmas 1-1.2

From Fig. 1, it is established that for the fish to reach its maturity level before 6 months, the initial population must be greater or equal to 6000 fish. Otherwise, the intrinsic growth rate has to be manipulated, whether this means better resources such as food, water, light, or something from an exogenous source that can enhance or hinder the rate at which the fish naturally reproduce. Nevertheless, with the hypothetical manipulation of the intrinsic growth rate, it is not possible to regulate the final value of the population.

### Simulation of a single-species population with coupling

For the second simulation, the fish population is coupled with an exogenous signal using model (8). Let *v* be a supposed number of fishermen or individuals between 1000 and 3000, who are trying to harvest as many fish as possible during the fishing season. The desire for this season is for the population to not be at risk of extinction when considering the maximum number of fishermen. By setting a control reference for the fishing season at $65\%$ of the maximum carrying capacity, the population will decrease to that reference at a minimum. When the fishing season is over, the control reference is changed to $90\%$ of *N*, which means that for both of the seasons the population will remain between $90\%$ and $65\%$ of its maximum carrying capacity.

**Fig. 2:**
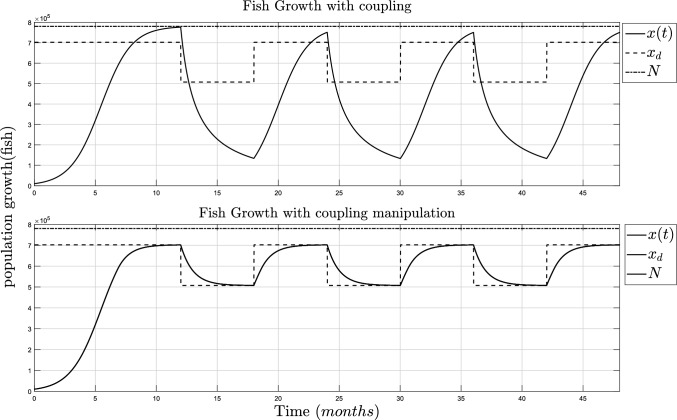
Simulation of the coupled Verhulst model for fish growth population. TOP: Population growth with maximum coupling parameters and individuals ($\alpha _0=-N/v$). BOT: Population growth with the control algorithm ($\alpha (t)$)

In the original research, Laham et al. (2012), a harvest function is introduced, *H*(*t*), from the data obtained during 6 months of harvesting. For this research, *H*(*t*) can be related to the term $\frac{r\alpha x(t)v}{N}$, where *x*(*t*) is the instant fish population, *r* is the intrinsic growth rate of the fish, *N* is the maximum carrying capacity of the fish, *v* is the exogenous source introduced to the fish population or the fishermen and $\alpha$ is the parameter related to the capacity at which every individual is allowed to harvest fish. From the analysis of the final value regulation, it is established that for the fish population to be exterminated, the maximum value of the coupling parameter has to be $\alpha >-N/v$, which will be denoted as $\alpha _0$. Using the parameters from Table 3, model (8) can be simulated when the coupling is at maximum capacity without risk of extinction and with different supposed numbers of individuals or fishermen. Although for the population to follow the control references set for each season, this parameter must be defined as manipulable, i.e., $\alpha :=\alpha (t)$, and then computed with Lemma 4. The simulation of the fish population’s growth with $\alpha _0$ and with $\alpha (t)$ for the fishing and growing season are shown in Fig. 2.

**Table 3:** Parameters of the Verhulst model with coupling signal

Parameter	Value	Units
*r*	0.8	Fish/t
*N*	780500	Fish
$\alpha _0$	−N/v	Fish/fishermen
*v*	1000	Fishermen
$x_0$	10000	Fish

**Fig. 3:**
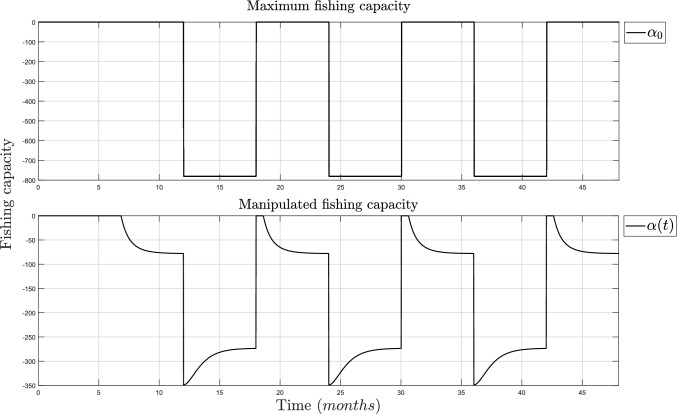
Simulation of the fishing capacity. TOP: Maximum coupling parameters and fishermen ($\alpha =-N/v$ and $v=1000$). BOT: Control algorithm ($\alpha (t)$)

Figure 2 (top) shows the fish growth is never at risk of extinction, but it does grow below its inflection point, thus making the fish not mature enough for proper harvesting. Meanwhile, in Fig. 2 (bot), the population regulation is shown and it follows the desired population value, $x_d$, which is defined between $65\%$ and $90\%$ of *N*. The simulation of the coupling parameters, $\alpha _0$ and $\alpha (t)$, is shown in Fig. 3.

On the top side of Fig. 3, the parameter changes between zero and $\alpha _0$ according to the harvest and growing season. On the bottom side of Fig. 3, it shows that the fish can be harvested for a time frame during the growing season but in a controlled capacity instead of avoiding any activity.

**Fig. 4:**
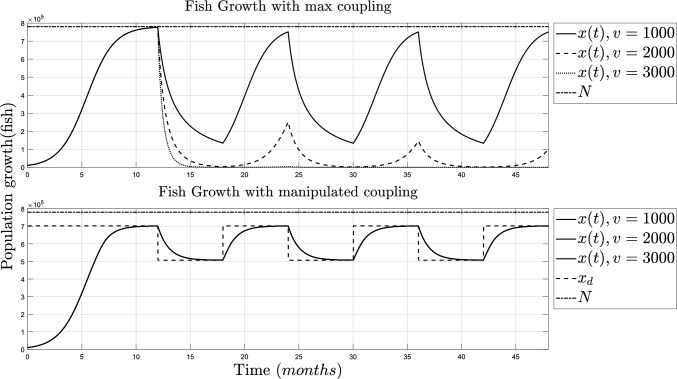
Simulation of the fish growth with coupling signal. TOP: Fish growth with Maximum coupling parameters ($\alpha _0=-N/v$ and $v=1000$). BOT: Fish growth using regulation Lemma 4 ($\alpha (t)$) for $v=1000$, $v=2000$, and $v=3000$

One of the advantages of representing the harvest function with the variable *v* and its coupling parameter $\alpha$ is the possibility of simulating scenarios with a different number of individuals and how the situation can be approached to prevent extinction. This simulation is done when there is an overpopulation of $v=2000$ and $v=3000$, the results can be seen in Fig. 4.

**Fig. 5:**
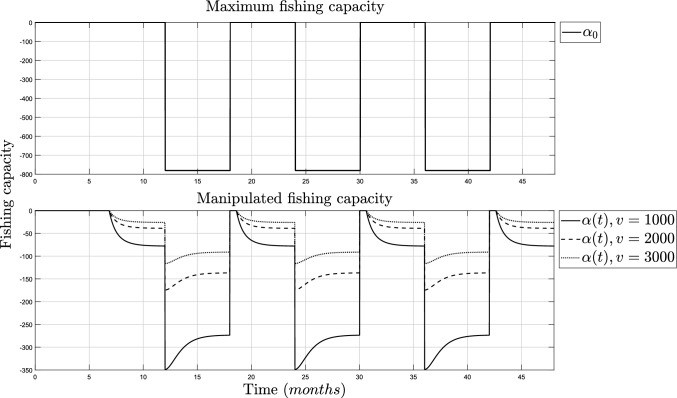
Simulation of the fishing capacity. TOP: Maximum coupling parameters and fishermen ($\alpha =-N/v$ and $v=1000$). BOT: Manipulated coupling parameter ($\alpha (t)$) for $v=1000$, $v=2000$ and $v=3000$

On the top side of Fig. 4, the fish population is shown when the coupling parameter is at the highest capacity, using $\alpha _0$, and it can be seen that the population goes to extinction when *v* is increased. On the bot side of Fig. 4, the fish population is shown with the manipulated coupling parameter, $\alpha (t)$, and the variation of *v* is not meaningful in the overall growth. The simulation of the parameters $\alpha _0$ and $\alpha (t)$ for all values of *v* is shown in Fig. 5.

On the bot side of 5, it can be seen that the fishing capacity is significantly reduced when the number of fishermen increases. The manipulation of the coupling parameter could be helpful in decision-making, for extinction prevention, and when a specific regulation is needed.

### Simulation of a double-species population

For the third simulation, the coexistence of two species is considered when *v*, previously seen as the number of fishermen, is now represented as a logistic equation, $v:=x_2(t)$, where the maximum capacity of fishermen is $N_2$, and their intrinsic growth rate is $r_2$. One assumption is that if the fishermen are seen as the clients of a fishing business, their parameters are governed by actions such as investment, maintenance, marketing, cost, promotions, discounts, and publicity. Therefore, the simplest representation of the fish population’s growth coupled with the fishermen population using model (15) is in the regime of Amensalism, where the fishermen grow independently of the fish, and the fish population can be regulated by the manipulation of the fishermen’s fishing capacity.

The simulation is approached as follows: For the fishermen, $x_2(t)$, the maximum carrying capacity is supposed as $N_2=5000$, the initial condition as $x_{02}=500$, and the objective for the population is set to grow from $x_{02}$ to $N_2$ in a total of 5 years to be in accordance with the previous simulation. The intrinsic growth rate, $r_2$, is calculated from Eq. (6) using 60 months as the transitory time duration. For the fish, $x_1(t)$, the parameters and initial condition are the same as in the previous subsection, and the control reference is now set between $45\%$ of $N_1$ for the fishing season and $65\%$ of $N_1$ for the growing season. This regulation is accomplished when the coupling parameter is defined as manipulated, $\alpha _1:=\alpha _1(t)$, and computed using Lemma (5). The simulation of the independent fishermen and its coupling with the fish population was done using the parameters of Table 4.

**Table 4:** Parameters of the coexistence logistic growth model representing Amensalism

Parameter	Value	Units
$r_1$	0.8	Fish/t
$r_2$	0.1166$^{1}$	Fish/t
$N_1$	780500	Fish
$N_2$	5000	Fishermen
$\alpha _{01}$	$-N_1/N_2$	Fish/fishermen
$\alpha _{02}$	0	Fishermen/fish
$x_{01}$	10000	Fish
$x_{02}$	500	Fishermen

$^{1}$Values obtained from Eq. (6)

**Fig. 6:**
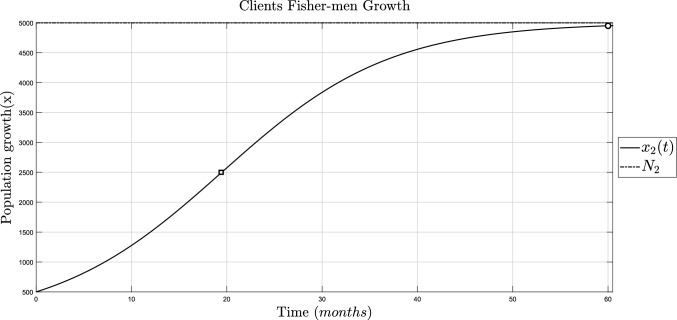
Simulation of the fishermen’s independent growth, $x_2(t)$, with parameters from Table 4

The fishermen’s growth is shown in Fig. 6, it reaches the $99\%$ of its maximum carrying capacity at 60 months and has an inflection point of 2500 individuals at 19 and a half months.

**Fig. 7:**
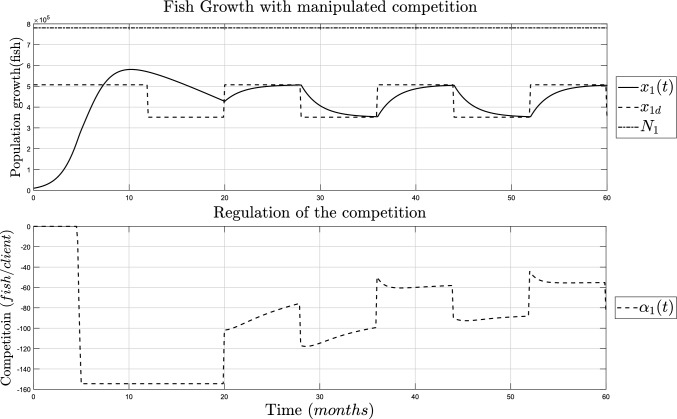
Simulation of a fish population coupled with a fisherman population. TOP: Growth of fish population with controlled fishing capacity (continuous line). BOT: Manipulation of the fishing capacity $\alpha _1(t)$ (dashed line)

Both populations are coupled during fishing and growing seasons, and the desired regulation is done with the manipulation of the coupling parameter. This is shown in Fig. 7, on the top side is the growing fish population coupled with the fishermen population shown in Fig. 6, and on the bottom side is the manipulation of the coupling parameter or fishing capacity using Lemma 5.

The capacity at which every individual is allowed to fish is adjusted depending on the fishermen’s growth, this is helpful for decision-making with an increasing number of individuals or clients. As it can be seen, for the first fishing season, the fish population tries to reach the regulation reference but it cannot since $\alpha (t)$ is at maximum value to prevent extinction. This simulation is established from its simplest interaction, where the fishermen cannot decrease, but with the appropriate definitions, they can be seen as a predator and the amount of fish as the prey, and have a similar objective of regulation where both of them coexist without risk of extinction.

## Conclusion

The mathematical models with Verhulst-like structure presented in this paper highlight three parameters: the intrinsic growth rate, the coupling parameter, and the maximum carrying capacity, and the latter one is considered known for this paper so the importance of the first two is presented. The analysis of these models was helpful for the constant computing of the intrinsic growth rate, the characterization of the coupling parameters, and the simulation of the models. The intrinsic growth rate can slow or speed up the process regarding time, but it is not able to modify the final value of the system.

The analysis of the single-species model is comparable with the analysis made by Tsoularis and Wallace, nevertheless, the research done by them is only for the modeling of one species, population, or growth variable. The results shown in this research include the analysis of a two-species Verhulst-like model and a special case considering one species with external coupling to find a regulation scenario.

These results show that the Verhulst-like structure for modeling and regulation purposes is really interesting. The regulation of a population is achieved through the manipulation of the coupling parameter only for three of the ecological regimes of species interaction. With the analysis of the final value of the models, Lemmas for population regulation were proposed for those ecological regimes.

The competition, amensalism, and predation regimes have the right structure for the regulation of its population between zero and its maximum carrying capacity. The results of a fish population and its interaction with human beings show that this regulation is possible. Besides, it could be helpful for simulation, preventing the extinction of a population and for decision-making purposes.

Cabella and Riveiro also tried to control the model, but it was focused on the maximum carrying capacity, stating that an external source or signal has a direct impact on this parameter. For this research, the coupling parameter was the one considered manipulable.

The limitation of this research’s design is that it can only be done with the right model structure. If the coupling parameter has a positive value it cannot be regulated and the model’s stability becomes a problem. A major benefit is that the analysis is done on the general mathematical model, meaning that it can be used for many different real-case applications that fit the stated structure. Furthermore, if any of the growth variables are unknown, then it would not be possible to use this methodology, but the future challenge is to predict or estimate these values and design the regulation with estimated variables.
